# Colchicine in Cardiovascular Disease: Evidence Structure, Clinical Efficacy, Safety, and Translational Positioning Across Cardiovascular Syndromes

**DOI:** 10.3390/ijms27104419

**Published:** 2026-05-15

**Authors:** Hossein Omidian, Luigi G. Cubeddu, Erma J. Gill, Luigi X. Cubeddu

**Affiliations:** 1 Department of Pharmaceutical Sciences, Barry and Judy Silverman College of Pharmacy, Nova Southeastern University, Fort Lauderdale, FL 33328, USA; omidian@nova.edu (H.O.); eg1262@mynsu.nova.edu (E.J.G.); 2 College of Osteopathic Medicine, Nova Southeastern University, Fort Lauderdale, FL 33328, USA; lcubedd0@mynsu.nova.edu

**Keywords:** colchicine, cardiovascular inflammation, coronary artery disease, atherosclerotic cardiovascular disease, acute coronary syndrome, secondary prevention, translational medicine

## Abstract

Colchicine has emerged as a prominent anti-inflammatory candidate in cardiovascular medicine, supported by a hierarchy of evidence spanning chronic and acute coronary syndromes, post-myocardial infarction care, revascularization, atrial fibrillation, pericardial disease, heart failure, peripheral arterial disease, and mechanistic translational models. Across this literature, the most mature study architecture and the strongest clinical support are derived from completed randomized trials in chronic coronary disease and secondary prevention, where colchicine has been shown to prevent major cardiovascular events (MACEs) when added to standard of care. The clearest clinical benefits are the reduction in non-fatal ischemic events in atherosclerotic disease, prevention of recurrent pericarditis and postoperative atrial fibrillation, and attenuation of inflammatory and plaque-related markers. By contrast, mixed or lower-tier evidence renders its application less consistent in acute coronary syndromes, ST-elevation MI (STEMI), percutaneous coronary intervention (PCI)-related hard outcomes, and heart failure, while a definitive mortality benefit has not been demonstrated. Overall, colchicine is best understood as a targeted clinical adjunct whose value depends heavily on precise indication, timing, dose, gastrointestinal tolerability, and the maturity of the supporting evidence.

## 1. Introduction: Framing the Role of Colchicine in Cardiovascular Care

**Scheme 1:**
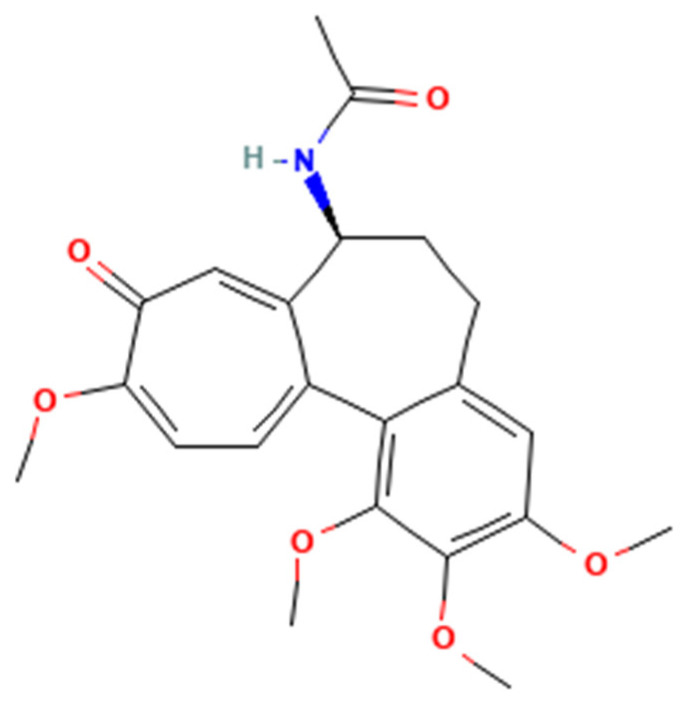
Colchicine structure [4].

Low-grade, persistent inflammation is now understood to be a central component of cardiovascular disease biology rather than a secondary epiphenomenon. Mechanistically, in atherosclerotic disease, inflammatory signaling directly participates in plaque evolution, destabilization, thrombogenicity, and recurrent ischemic risk; in parallel, procedure-related inflammation contributes to myocardial injury, arrhythmia, and pericardial or perioperative complications. This conceptual shift has created a translational imperative: to identify anti-inflammatory therapies that are mechanistically relevant, clinically deployable, and scalable across cardiovascular practice [1,2,3]. Within this landscape, colchicine has emerged as one of the most intensively studied anti-inflammatory candidates (Scheme 1). Its potent anti-inflammatory actions led to U.S. Food and Drug Administration (FDA) approval in 2009 for the treatment of familial Mediterranean fever and acute gout flares, providing the historical foundation for subsequent randomized trials that led to its 2023 approval for the treatment of adult patients with established atherosclerotic cardiovascular disease (ASCVD) or multiple cardiovascular disease risk factors [4]. In cardiovascular medicine, colchicine’s appeal lies not only in its established anti-inflammatory pharmacology in chronic ASCVD, but in the breadth of primary cardiovascular settings in which it has been investigated. The clinical and observational literature now spans chronic and acute coronary syndromes (ACS), post-myocardial infarction (MI) care, percutaneous coronary intervention (PCI) and surgical revascularization, atrial fibrillation and ablation-related inflammation, pericardial disease, inflammatory cardiomyopathy, heart failure, and peripheral arterial disease (PAD) [5,6,7,8,9,10,11,12,13,14,15,16]. This expansion of interest reflects a broader hypothesis, supported by both mechanistic data and clinical trials, that colchicine may influence cardiovascular risk not through a single disease-specific mechanism, but through shared inflammatory pathways operating across vascular injury, myocardial remodeling, plaque biology, and periprocedural stress [2,3,14,17,18].

The field, however, is no longer defined simply by the foundational question of whether colchicine “works.” Based on the current hierarchy of evidence, the more important translational problem is how to position colchicine within cardiovascular therapeutics with precision. That problem includes identifying the specific clinical contexts in which inflammation is most likely to be a modifiable risk, carefully distinguishing the strong evidence base in chronic secondary prevention from the less consistent data in acute periprocedural or post-MI applications. It also requires clarifying whether colchicine acts primarily through inflammasome-linked pathways or through a broader network of mechanistic effects involving neutrophil activation, platelet-thrombo-inflammatory signaling, cytoskeletal regulation, and plaque-level biology [17,18,19,20,21,22,23]. It also requires attention to implementation-level questions—delineated by ongoing and completed clinical studies—including treatment timing, dose, exposure duration, patient selection, drug and disease interactions, and the relationship between mechanistic plausibility and clinically meaningful therapeutic use [8,10,11,13,17,24].

This review frames the field around a unifying translational question: whether colchicine can be integrated as a targeted anti-inflammatory strategy across distinct but biologically related cardiovascular syndromes, and what mechanistic, observational, and randomized clinical logic supports that integration [2,3,8,10,13,16,17]. This review, therefore, situates the evidence within that broader framework, emphasizing the biological rationale, the evolution of the clinical investigation agenda, and the key translational challenges that continue to shape the field.

## 2. Trial Methodologies and Study Setup

The clinical and translational evidence evaluating colchicine in cardiovascular disease is structurally broad, covering a wide range of study designs. However, the rigor and maturity of this evidence base are unevenly distributed. The most robust data—comprising large, randomized, placebo-controlled, multicenter trials—are concentrated in chronic coronary artery disease (CAD) and post-myocardial infarction (MI) populations. Conversely, the application of colchicine in non-CAD indications is predominantly supported by lower-tier evidence, including smaller observational cohorts, pilot studies, crossover designs, and mechanistic investigations. Furthermore, the broader literature base includes substantial retrospective matched analyses, nested sub-studies within major randomized controlled trials (RCTs), pooled meta-analyses, predictive modeling studies, and a vast array of translational literature spanning ex vivo, in vitro, and animal models.

### 2.1. Clinical Populations and Trial Structures: A Hierarchy of Evidence

As detailed in Table 1, the evaluated clinical populations span a broad cardiovascular spectrum. This includes acute coronary syndromes (ACS), recent and remote MI, chronic or stable CAD, percutaneous coronary intervention (PCI), coronary artery bypass grafting (CABG), atrial fibrillation ablation, recurrent pericarditis, acute and chronic heart failure, peripheral arterial disease (PAD), aortic stenosis, and inflammatory cardiomyopathy. To clarify the level of evidence, it is essential to distinguish between the various trial structures employed:

**Major Completed and Ongoing Randomized Controlled Trials (RCTs):** The architecture of these studies represents the highest level of clinical evidence, though they vary in scope from targeted mechanistic studies to massive international event-driven outcome trials. Representative examples include:

- **Completed Post-MI/Chronic CAD Trials:** The landmark LoDoCo2 trial enrolled 5522 patients with chronic CAD [45]. The CLEAR SYNERGY trial, a 2 × 2 factorial study, included 7062 post-STEMI patients across 104 centers in 14 countries [10]. The COLCOT enrolled 4745 patients within 30 days of an acute MI [73]. The COPS trial in 795 patients with colchicine started during an MI [74]. The COVERT-MI multicenter randomized, double-blind STEMI program enrolled 194 patients (with follow-up reported in 192) [8,75]. A prospective randomized double-blind ACS trial enrolled 249 patients with a 6-month follow-up [76].
- **Completed Procedural/Electrophysiology Trials:** The multicenter randomized PAPERS trial evaluated 139 post-ablation patients [77].
- **Completed Targeted Phenotype Trials:** The ViKCoVaC double-blind, 2 × 2 factorial trial enrolled 154 patients with diabetes and coronary calcification (149 completed a 3-month evaluation) [65].
- **Ongoing/Planned RCTs:** Significant ongoing efforts include the planned international COP-AF trial targeting 3200 surgical patients across 40 sites in 11 countries [9], the event-driven COL BE PCI trial aiming for 2770 PCI patients [11], and the COLICA multicenter randomized double-blind acute heart failure trial targeting 278 patients across 12 sites [15,24].

**Figure 1:**
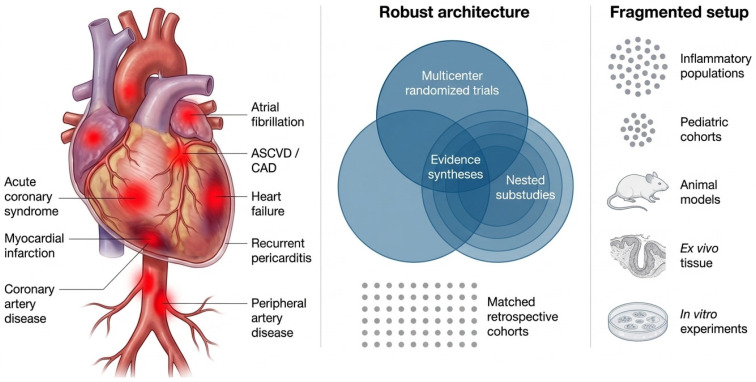
**Conceptual map of populations and study architectures in the colchicine cardiovascular literature.** The figure summarizes the principal clinical populations represented in this section—centered on ACS, MI, CAD, atrial fibrillation, recurrent pericarditis, heart failure, and PAD—and contrasts the most methodologically robust evidence structures, such as multicenter randomized trials, matched retrospective cohorts, evidence syntheses, and nested sub-studies, with more fragmented settings represented by inflammatory populations, pediatric cohorts, animal models, ex vivo tissue studies, and in vitro experiments.

As illustrated in Figure 1, the density and methodological robustness of these populations firmly establish coronary and post-infarction settings as the primary domains of high-quality evidence.

### 2.2. Observational Cohorts and Nested Sub-Studies

Supplementing the RCT data is a substantial body of non-randomized, observational evidence (Table 1). While lower on the evidence hierarchy, these studies—comprising retrospective comparative cohorts, matched analyses, and pharmaco-epidemiology—provide critical insights into real-world application and targeted subpopulations.

Key observational examples include:

- A TriNetX retrospective TAVI analysis identifying 52,860 patients, matching 705 colchicine-exposed patients with 702 controls (1- and 6-month follow-up) [45].
- A propensity score-matched study of 1568 atrial fibrillation ablations in 1412 patients (275 matched patients per group; mean follow-up 34 months) [37].
- A retrospective, multicenter cohort of recurrent pericarditis patients followed for 12 months [66].
- A double-center retrospective myopericarditis cohort of 175 patients (73 matched pairs; median 25.3-month follow-up) [46].
- A target trial emulation in 1820 Medicare beneficiaries with peripheral artery disease and gout over 2 years [78].
- A retrospective real-world acute MI (AMI) cohort of 1796 patients [79].
- A Dutch nationwide pharmaco-epidemiologic study of 84,582 gout patients [80].
- A large propensity score-matched PAD cohort comparing 52,350 pairs over 10 years [47].

Furthermore, important nested sub-studies within major parent RCTs have refined the population landscape, offering deeper internal characterization. These include LoDoCo2 analyses of CT imaging, biomarkers, diabetes, clonal hematopoiesis, and extracellular vesicles [25,26,27,28,73]; pharmacogenomic work in 1522 COLCOT participants [29]; and pooled patient-level post-MI inflammatory analyses from COLCOT and LoDoCo-MI [30], as well as from the CLEAR-SYNERGY study [81,82,83].

### 2.3. Evidence Syntheses and Modeling Studies

A large proportion of the literature consists of evidence syntheses and predictive modeling, predominantly focused on patients with coronary or vascular disease.

**Meta-Analyses:** Pooled analyses form the structural breadth of the dataset, consolidating data from various RCTs. Representative syntheses include meta-analyses of 16 randomized trials (20,601 patients) [5], 21 RCTs (14,188 patients) [52], 11 RCTs (12,869 patients) [84], 10 randomized trials (22,532 patients) [7], 9 randomized trials (30,659 patients) [53], 14 randomized trials (31,397 participants) [13], 11 randomized trials (30,808 participants in ASCVD) [16], and the most recent, including 6 RCTs, with a pooled sample size of 21,800 patients [85]. More focused syntheses have addressed specific domains: recurrent pericarditis [12], postoperative atrial fibrillation [54,86], recent MI [55], ACS [56,57,87], PCI populations [88,89], general CAD [90], and chronic CAD [91,92].

**Modeling Studies:** Modeling frameworks extend beyond actively enrolled cohorts to project outcomes and cost-effectiveness. These include probabilistic or Markov-based analyses derived from Canadian post-MI data [93], LoDoCo2 [94], 27,443 U.S. veterans after CABG [95], COLCOT’s 4745 randomized post-MI patients [96], national U.S. prescription and survey data in stable CAD [97], and SMART-REACH modeling across 36,642 individuals from LoDoCo2, UCC-SMART, and REACH [98].

Table 1 summarizes the major cross-study structural patterns, grouping recurring design architectures and quantitative study setup markers.

### 2.4. Preclinical, Ex Vivo, and Translational Models

The foundational basis for colchicine’s cardiovascular application rests on a substantial translational and experimental literature (Table 1), which elucidates the mechanistic rationale underpinning its clinical deployment.

- **Preclinical In Vivo Models:** These include porcine MI (27 pigs) [58], rabbit abdominal aortic atherosclerosis (20 animals) [59], rat coronary microembolization (40 Sprague–Dawley rats) [60], murine post-MI models (adult C57BL/6 mice; 32/group) [61], ApoE-/- aneurysm models [99], elastase/3-aminopropionitrile abdominal aortic aneurysm models (eight-week-old male C57BL6/J mice; 28 treated/29 control; 80-day follow-up) [62], CVB3 myocarditis in mice [100], sterile pericarditis rat models [63], doxorubicin-induced dilated cardiomyopathy models [101], and rat or mouse ischemia–reperfusion studies linked to STEMI patient analyses [102].
- **Ex Vivo and In Vitro Work:** This includes living myocardial slices perfused for 90 min [20], platelet aggregation studies in 35 chronic coronary syndrome patients (including 7 clopidogrel non-responders) [19], oxLDL-stimulated T-cell assays [103], cholesterol-crystal bench studies using in vitro and human plaque material [104], ex vivo human carotid plaque exposure experiments [23], and PCI-linked neutrophil studies in 60 patients with accompanying in vitro analyses in neutrophils from 10 ACS patients [22].

Collectively, the evidence base spans randomized, blinded, matched, crossover, factorial, multicenter, event-driven, observational, imaging-based, ex vivo, animal, and modeling designs across a wide range of cardiovascular and inflammatory populations.

The principal limitation of this study-setup evidence is its marked heterogeneity across disease settings, design architectures, and levels of evidence. Randomized coronary secondary-prevention trials coexist alongside retrospective matched cohorts, pilot procedural studies, case reports, modeling analyses, and preclinical experiments. This heterogeneity constrains direct comparability and weakens the uniform assessment of external validity [20,21,31,45,48,66,67,93,105].

Furthermore, the evidence is unevenly distributed: large, mature datasets are concentrated in populations with chronic CAD, ACS, and post-MI. In contrast, inflammatory cardiomyopathy, heart failure with preserved ejection fraction (HFpEF), pediatric postoperative settings, recurrent pericarditis subgroups, and several mechanistic contexts remain represented mainly by small or selective cohorts [6,14,15,24,67,68,69,70,71]. In addition, many publications represent secondary, post hoc, or biomarker analyses of the same parent trials (particularly LoDoCo2, COLCOT, CLEAR, and COVERT-MI), which deepens internal characterization but limits the breadth of unique clinical sampling [10,26,27,28,30,32,33,34,106,107].

Future work must prioritize new, independent multicenter cohorts in underrepresented phenotypes. There is a need for more consistent reporting of eligibility structures and follow-up windows, and the prospective integration of mechanistic imaging and biomarker platforms within adequately powered clinical studies, rather than relying on isolated nested analyses [6,9,11,17,18,24,25,27,35,50].

Overall, the population and design literature is broad and technically rich. However, its maturity remains concentrated in coronary secondary-prevention settings, while several other clinically relevant domains still rely on smaller, fragmented, or translationally oriented study structures [7,10,13,15,16,24,33,54].

## 3. Intervention and Outcome Framework

**Figure 2:**
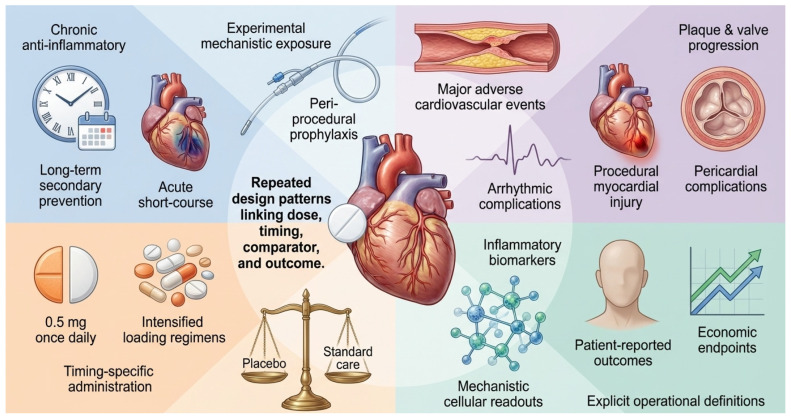
**Overview of the intervention and outcome framework of colchicine studies.** The figure summarizes the recurring study designs in the literature, linking colchicine dose and timing, comparator structure, and major outcome domains, including cardiovascular events, procedural and arrhythmic complications, imaging progression, biomarkers, mechanistic readouts, patient-reported outcomes, and economic endpoints.

This section focuses on the intervention/exposure structure and outcome architecture of the included studies. It identifies which colchicine regimen or exposure was evaluated, against which comparator, and with which endpoints, operational definitions, and assessment timepoints. (Note: this section excludes sample structure, effect estimates, effect direction, and interpretation, focusing solely on the design of the intervention and outcome assessment.) As summarized visually in Figure 2 and Table 2, the literature is organized around repeated design patterns linking dose, timing, comparator choice, and outcome domain to the specific cardiovascular context under study.

### 3.1. Colchicine Regimens and Comparators

Across the included studies (Table 2), colchicine was evaluated in several recurring therapeutic configurations: periprocedural prophylaxis, short-course therapy around acute ischemic or inflammatory events, longer-term secondary prevention, chronic anti-inflammatory treatment, and mechanistic exposure in ex vivo, in vitro, and animal models.

**Standard and Intensified Regimens in Clinical Trials:**

- **Chronic Use:** In cardiovascular clinical studies, the most common regimen was low-dose colchicine at 0.5 mg once daily, used in chronic CAD, post-MI, ACS, and plaque-imaging studies, typically against placebo or standard care [2,7,10,11,14,17,18,33,34,50].
- **Periprocedural/Acute Use:** Shorter intensified regimens were also frequent, including:
  ○ A total of 0.6 mg twice daily for 7 days after radiofrequency ablation [77].
  ○ A total of 0.6 mg twice daily for 10 days after major thoracic surgery [9].
  ○ A total of 0.6 mg twice daily for 14 days after AF ablation [43].
  ○ A total of 2 mg loading followed by 0.5 mg twice daily for 5 days in STEMI reperfusion trials [8,51].
- **PCI-Specific Strategies:** Other PCI-related strategies included:
  ○ A total of 1 mg then 0.5 mg given 6–24 h before PCI [40].
  ○ A single 1.8 mg preprocedural dose [44].
  ○ A total of 1 mg before PCI followed by 0.5 mg daily until discharge [39].
  ○ A total of 0.6 mg daily initiated within 24 h after PCI, with continuation or discontinuation at 1 month determined by hs-CRP levels [112].
- **Heart Failure and Cardiomyopathy:** Reported regimens included 0.5–1.0 mg daily in chronic inflammatory cardiomyopathy [6], 2 mg loading then 0.5 mg twice daily for 8 weeks in acute heart failure [24], and 0.5 mg twice daily for 12 weeks in HFpEF or HFrEF trials [71,117].

**Timing-Based Exposure and Comparators:** Some studies defined exposure by timing rather than a fixed schedule. Examples include colchicine use within 3 months before TAVI [45]; initiation <3 days, 4–7 days, or >8 days after MI [32]; or administration from 7 days before to 1 month after AF ablation (versus post-ablation only or no colchicine) [108].

Comparators were most often placebo, standard care, usual care, or no colchicine. However, several studies used active comparators or combination strategies, including NSAIDs, atorvastatin alone, spironolactone, vitamin K1, anakinra alone, intrapericardial steroid instillation alone, amiodarone, extracellular vesicles, and intensive LDL-C-lowering therapies [63,65,66,106,107,113,118].

**Experimental Exposures:** Experimental studies utilized concentration-based or weight-based colchicine exposure. These included 10 μM in platelet and T-cell assays [19,103], 25 nmol/L in neutrophil studies [22], 2 or 10 ng/mL for 24 h in human plaque sections [23], and 0.1–0.3 mg/kg/day in murine or rat cardiovascular injury models [60,61,64,101,119,120].

### 3.2. Outcome Assessment Frameworks

Outcome selection across the literature was highly context-specific but followed a consistent structural logic spanning clinical events, imaging surrogates, biomarker endpoints, and economic models.

**Clinical Event Endpoints:**

- **Coronary and Post-MI:** The predominant endpoints were Major Adverse Cardiovascular Events (MACEs) or related composite ischemic outcomes, typically comprising combinations of cardiovascular death, MI, stroke, urgent hospitalization for angina, ischemia-driven or repeat revascularization, cardiac arrest, and all-cause mortality [8,10,11,13,16,32,34,36,55,57,84,91,92].
- **Arrhythmia and Procedure-Focused:** Endpoints included clinical pericarditis within 14 days after ablation [77], AF recurrence (operationalized in one study as detection >30 s after a 3-month blanking period) [37], perioperative AF/flutter and myocardial injury after noncardiac surgery (MINS) [9], postoperative AF [54,86,109,121], no-reflow, periprocedural myocardial injury, coronary microvascular dysfunction, coronary flow reserve, resistive reserve ratio, constrictive physiology at 1 and 4 weeks after open-heart surgery, and post-procedural chest pain, pericardial effusion, emergency visits, and hospitalization for recurrence [39,40,41,43,110].
- **Pericardial and Myocarditis:** Measured outcomes included recurrence incidence, treatment failure, rehospitalization, and event-free survival [12,46,66,100,101,122].
- **Peripheral and Vascular Studies:** These assessed major adverse limb events, amputation, limb revascularization, aortic enlargement, aneurysm incidence, and heart disease or ischemic cardiovascular disease risk in observational cohorts [47,49,62,72,80,99,116].

**Imaging, Biomarker, and Functional Surrogates:** Several studies utilized imaging and functional surrogates to define disease progression or inflammatory activity.

- **Imaging:** Endpoints included infarct size by cardiac magnetic resonance at 5 days and 3 months, microvascular obstruction, left ventricular ejection fraction (LVEF), left ventricular remodeling, global longitudinal strain, carotid–femoral pulse wave velocity, coronary maximum tissue-to-background ratio on F-18-NaF PET, low-attenuation plaque volume and total atheroma volume on coronary CT-angiography (CCTA), aortic valve calcium score, 18F-NaF uptake, mitral annular calcification, and peak aortic jet velocity [6,14,17,18,50,65,75,123,124,125].
- **Biomarkers and Mechanistic Readouts:** A substantial subset of studies focused on hs-CRP, IL-6, IL-1β, IL-10, IFN-γ, TNF-α, white blood cell and neutrophil counts, NT-proBNP, sST2, urine albumin-to-creatinine ratio, circulating microRNAs, extracellular-vesicle NLRP3, serum GPVI, platelet reactivity in PRU or ARU, aspirin and clopidogrel resistance, NET release, tubulin organization, tissue factor expression, FXa generation, pyroptosis-related markers, AMPK/SIRT1/NLRP3 signaling, NOX2/ROS and Ca^2+^ influx, S100A8/A9 signaling, PAD4-related activity, macrophage polarization, autophagy-related readouts, collagen degradation, myofibroblast activation, and cholesterol-crystal morphology [19,20,21,22,23,24,28,29,30,42,60,71,102,103,104,112,113,114,115,119,120,126,127]. Also assessed were plasma CRP, cardiac biomarkers, electrocardiographic indices of atrial activation, ventricular function, and fibrosis-related markers [12,46,66,100,101,122].

**Operational Definitions, Safety, and Economic Modeling:** Operational definitions provided precise thresholds for evaluation. Examples included a PVC burden increase ≥50% and LVEF reduction >10% within a 6-month composite endpoint in inflammatory cardiomyopathy [6]; hs-CRP ≥ 2 mg/L defining high inflammation and hs-CRP ≤ 1.0 mg/L as a target threshold after MI [30,114]; and BARC type 3/5 bleeding within a 12-month net adverse clinical events composite after PCI [112].

Safety and tolerability outcomes included gastrointestinal events, diarrhea, myalgias, drug discontinuation, infection, pneumonia, sepsis, serious adverse events, and major bleeding. Patient-centered outcome frameworks included EQ-5D-5L, Seattle Angina Questionnaire, and HDRS-21 over varying follow-up periods [9,15,52,54,68,87,112,117,128,129,130]. Economic and modeled studies utilized quality-adjusted life-years, costs, net monetary benefit, projected event prevention, lifetime gain in MACE-free years, and long-horizon cost-effectiveness estimates rather than direct clinical endpoints [93,94,95,96,98]. Table 2 summarizes the principal cross-study design patterns that recur across these intervention and endpoint structures.

### 3.3. Limitations and Harmonization Needs

The intervention-and-outcome literature is broad but methodologically uneven. Colchicine schedules range from single preprocedural loading doses to 5–14-day periprocedural courses and long-term 0.5 mg/day prevention strategies. Concurrently, outcome frameworks vary from conventional cardiovascular composites to imaging surrogates, inflammatory biomarkers, arrhythmia endpoints, mechanistic laboratory readouts, and economic models. This extensive variability limits direct cross-study comparability at the design level [8,9,10,14,18,24,40,54] (Table 2).

Future studies would benefit from greater harmonization of regimen reporting and endpoint selection. Specifically, there is a need for clearer specification of dose, loading strategy, start time, duration, and discontinuation rules in timing-sensitive settings such as MI, PCI, and ablation [32,40,108,112]. Furthermore, core outcome sets must be developed that explicitly link surrogate measures to adjudicated clinical events across coronary, heart failure, peripheral vascular, and valve disease studies [6,14,17,47,71,78].

Taken together, this section demonstrates that colchicine has been tested through recurring design motifs—low-dose chronic therapy, short periprocedural exposure, and biomarker- or imaging-enriched assessment. However, to maximize comparability and clinical interpretability, the field now requires more standardized intervention architecture and integrated outcome hierarchies.

## 4. Findings: Efficacy, Mechanisms, and Safety

Across the full set of syntheses, colchicine showed its clearest benefits in reducing non-fatal ischemic events, selected arrhythmic and pericardial complications, and inflammatory or plaque-related markers. In contrast, mortality benefits were generally not demonstrated, and gastrointestinal intolerance was the most reproducible adverse effect. Table 3 summarizes the key cross-study evidence patterns.

### 4.1. Cardiovascular Efficacy in Coronary and Vascular Disease

When evaluating efficacy, a clear hierarchy of evidence emerges, distinguishing the robust benefits seen in stable secondary prevention from the more mixed results in acute post-infarction settings.

**Chronic and Established CAD (High-Tier Evidence):** In chronic coronary disease, the highest level of evidence—comprising multiple meta-analyses of completed randomized trials—showed significant reductions in primary outcomes defined as major adverse cardiovascular events (MACEs) and related non-fatal ischemic outcomes. Reported effect sizes ranged from a relative risk (RR) of 0.64 (95% CI 0.51–0.80, *p* < 0.01) to 0.83 (95% CI 0.73–0.95, *p* = 0.006) [7,16,84,85,91,92]. These benefits were driven mainly by lower rates of MI, stroke, and PCI. Specifically, the risk of MI was reduced in several pooled analyses from 17% to 36% [5,7,16,134]. Revascularization was likewise lower (17–27% risk reduction) [5,16,134], and stroke reduction was also recurrent in pooled evidence [57,85] (Table 3).

**Peripheral Arterial Disease (Observational Evidence):** In patients with PAD, large observational cohorts have reported that colchicine treatment is associated with lower major adverse limb and MACE outcomes. This included a 16% risk reduction for major adverse limb events (*p* = 0.002) and a 10% risk reduction for MACEs (*p* = 0.02) [116], alongside a reported hazard ratio (HR) of 0.90 (95% CI 0.88–0.92, *p* < 0.001) for the composite of cardiovascular and limb events [47].

**Acute MI and STEMI (Mixed Clinical Evidence):** The evidence supporting colchicine’s use immediately following an acute MI remains mixed. Some completed studies supported benefit, including fewer MACEs in one small, randomized trial (8/120 vs. 28/129, *p* = 0.001) [76], and early post-MI treatment in the landmark COLCOT trial, which demonstrated an HR of 0.52 (95% CI 0.32–0.84) for the primary endpoint when initiated within 3 days [32,73,135]. Furthermore, a meta-analysis of two ACS trials showed lower composite events (5.5% vs. 7.6%; OR 0.67, 95% CI 0.46–0.98, *p* = 0.04), cerebrovascular events, and urgent revascularization [57]. However, other completed randomized trials—such as the COPS and CLEAR-SYNERGY trials—and subsequent meta-analyses found no significant reduction in MACEs when initiated after a recent MI. This null data includes an RR of 0.83 (95% CI 0.66–1.04) within 1 month of MI [55], no significant reduction in pooled MACEs or mortality endpoints in another meta-analysis [136], and a lack of benefit in specific STEMI reperfusion trials [51,131]. However, the short duration of the trial and the use of all-cause mortality rather than cardiovascular mortality contributed to the lack of significant benefits observed in the COPS trial [74].

Several confounding, as well as trial conduct and item factors, were identified that may explain the lack of colchicine efficacy observed in the CLEAR-SYNERGY trial. Among them, a large loss to follow-up, non-adherence, and under-reporting associated with the COVID-19 pandemic affected the value and interpretation of the findings (HR 0.99 (0.85–1.16); *p* = 0.93) [132]. Additionally, an upward increase in the number of pericarditis cases in the colchicine group, a poor anti-inflammatory response to colchicine, as judged by hsCRP levels >3 mg/L in the colchicine-treated group, and an unusually large number of participants (*n* = 3000) at Macedonian and Serbian sites may have also influenced the neutral effects of colchicine [81,82,83].

Overall, the strongest efficacy signal is therefore established for non-fatal recurrent ischemic events in chronic or stabilized vascular disease, rather than for acute MI mortality or broad post-PCI event reduction [5,7,16,32,47,55,57,74,84,91,92,116,132,134].

### 4.2. Arrhythmic, Pericardial, and Perioperative Outcomes

Colchicine also demonstrated important clinical benefits across several inflammatory and rhythm-related settings, though efficacy varied by indication.

- **Postoperative Atrial Fibrillation (Strong RCT Evidence):** In postoperative AF prevention, pooled data showed a meaningful 35% risk reduction [54], a 46% risk reduction after CABG [121], and a 48.5% risk reduction with CABG or aortic valve replacement [109].
- **Pericardial Disease (Strong RCT/Observational Evidence):** In recurrent pericarditis, colchicine reduced recurrence (RR 0.46, 95% CI 0.37–0.58), treatment failure (RR 0.42, 95% CI 0.31–0.57), and rehospitalization (RR 0.26, 95% CI 0.10–0.70) [12]. In myopericarditis cohorts, recurrence was lower at 19.2% versus 43.8% (*p* = 0.001), with longer event-free survival (*p* = 0.005) [46]. Furthermore, in anakinra-treated patients with colchicine-resistant recurrent pericarditis, colchicine was still associated with lower recurrence (18.8% vs. 31.3%, *p* = 0.036) [66].
- **AF Ablation (Mixed/Null Evidence):** Results following AF ablation were less uniform. Colchicine was more consistently effective for postoperative AF prevention and recurrent pericardial disease than for AF ablation rhythm control [108]. In contrast to surgical settings, randomized and retrospective ablation studies reported no reduction in short-term atrial arrhythmia recurrence or several post-ablation clinical endpoints [12,37,38,43,46,48,54,66,108,109,121] (Table 3).

### 4.3. Biomarker, Plaque, and Mechanistic Findings

Across the syntheses, a hierarchy of mechanistic, imaging, and biomarker findings strongly aligned with colchicine’s anti-inflammatory and plaque-stabilizing clinical effects.

**Clinical Biomarkers:** In clinical trial sub-studies, colchicine significantly reduced hs-CRP in meta-analytic evidence (*p* = 0.0001), a reduction associated with 22% fewer clinical events, although the direct correlation between CRP change and events was weak [133]. After an MI, colchicine increased the odds of reaching the target threshold of hs-CRP ≤ 1.0 mg/L by 36% [30]. In chronic CAD, it successfully lowered extracellular vesicle NLRP3 (median 1.38 vs. 1.58 ng/mL, *p* = 0.025) and hs-CRP (median 0.80 vs. 1.34 mg/L, *p* < 0.005) [28]. It also consistently reduced multiple inflammatory mediators in PCI-related and ACS settings, including IL-6, TNF-α, IFN-γ, white blood cell count, neutrophil counts, and myeloperoxidase [42,44,50] (Table 3).

**In Vivo Plaque Imaging:** Plaque-focused clinical imaging studies support direct biologic activity in the vasculature. Optical coherence tomography (OCT) in ACS patients showed thicker fibrous caps with colchicine (87.2 μm vs. 51.9 μm; difference 34.2 μm, *p* = 0.006), reduced lipid arc (−35.7° vs. −25.2°, *p* = 0.004), and less macrophage extension (difference −6.0°, *p* = 0.044) [50]. Coronary CT studies showed no significant change in low-attenuation plaque volume in one trial, but lower total percent atheroma volume and trends toward regression in non-calcified plaque [124]. Conversely, another study found increased calcified plaque volume and burden without a significant change in pericoronary adipose tissue attenuation [25].

**Figure 3:**
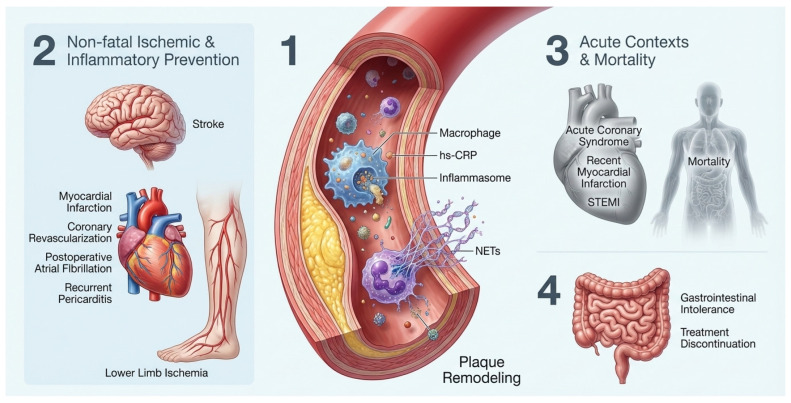
**Integrated schematic summary of the findings associated with colchicine across cardiovascular settings.** Central anti-inflammatory and plaque-modifying effects include attenuation of hs-CRP, inflammasome-related activity, macrophage and neutrophil extracellular trap pathways, and plaque remodeling. These biologic effects align with the most consistent clinical findings—reduction in non-fatal ischemic events, postoperative AF, recurrent pericarditis, and lower-limb ischemic complications—while benefit remains less consistent in ACS, recent MI, and STEMI, and gastrointestinal intolerance and treatment discontinuation remain the principal harms.

**Ex Vivo and Experimental Mechanisms:** Providing the foundational biologic rationale, additional ex vivo and experimental work demonstrated colchicine’s suppression of neutrophil extracellular traps (NETs), tissue factor expression, platelet aggregation pathways, cholesterol crystal volume, pyroptosis, fibrosis, and adverse remodeling [19,20,21,22,23,60,61,100,102,103,104,119,127]. These interrelated clinical and biological domains are shown in Table 3 and synthesized visually in Figure 3.

### 4.4. Safety and Harm

**Gastrointestinal Toxicity:** The dominant adverse effect across all syntheses was gastrointestinal toxicity. In a large meta-analysis of 21 completed RCTs, gastrointestinal events occurred in 16.1% (colchicine) versus 12.2% (control), diarrhea in 12.5% versus 8.1%, and study discontinuation in 4.8% versus 3.4% [52]. CAD-specific analyses similarly reported more gastrointestinal adverse events and discontinuation [84]. Individual studies repeatedly reproduced this pattern, including GI discomfort after AF-ablation prophylaxis (47% vs. 15%, *p* < 0.001) [77], GI symptoms after ACS treatment (12.5% vs. 2.5%, *p* = 0.002) [76], diarrhea after cardiac surgery (25.7% vs. 11.8%, *p* = 0.005) [109], and severe diarrhea leading to early discontinuation after high-power short-duration AF ablation [43].

**Mortality Signals and Pharmacologic Interactions:** Mortality signals were inconsistent and require cautious interpretation. Many pooled analyses showed no significant increase in cardiovascular or non-cardiovascular death [5,7,53,84]. However, some studies reported increased non-cardiovascular death, including an OR of 1.54 (95% CI 1.10–2.15, *p* = 0.01) [134] and an RR of 1.53 (95% CI 1.10–2.14, *p* = 0.01) [129]. The completed COPS trial found numerically fewer primary composite events but higher total death and non-cardiovascular deaths [74]. In a LoDoCo2 cause-specific analysis, cardiovascular death was 0.7% versus 0.9%, while non-cardiovascular death was 1.9% versus 1.3% [31]. The most recent meta-analysis showed neutral effects of colchicine on all-cause mortality and non-CV mortality in 21,800 patients with vascular disease [85].

Overall, the adverse-effect profile is best characterized as consistently gastrointestinal, with uncertain and non-uniform signals regarding non-cardiovascular mortality [31,43,52,74,76,77,84,85,109,129,134]. Crucially, due to its narrow therapeutic index, colchicine dosages must be reduced or interrupted in patients with normal renal and hepatic function if treatment with a P-gp inhibitor or a strong CYP3A4 inhibitor is required. Dose adjustments and contraindications should be considered in patients on antifungals (ketoconazole, itraconazole, voriconazole), clarithromycin, antivirals (ritonavir, saquinavir), amiodarone, verapamil, and diltiazem, among others. Additionally, colchicine should be avoided in advanced liver failure and in patients with renal insufficiency with glomerular filtration rates of ≤30 mL/min [137].

The collective findings must be interpreted in light of substantial heterogeneity across studies in population, timing, dose, duration, comparator, and endpoint construction, including wide variation across stable CAD, ACS, STEMI, PAD, pericarditis, AF ablation, surgical cohorts, and preclinical models [5,32,54,55,78] (Table 3).

Several areas of apparent benefit or inconsistency rely heavily on secondary, subgroup, sensitivity, or post hoc analyses rather than primary prospective confirmation. This is particularly true for evaluating the timing of treatment initiation, biomarker-defined effect modification, and regional or baseline-risk differences [26,27,32,33,34,35,36,138].

Future work must therefore prioritize harmonized, adequately powered randomized trials that can define the optimal initiation window, dose, and duration in ACS settings. It is necessary to distinguish clearly between procedural contexts with repeated benefit and those with repeated null results. Furthermore, ongoing trials should prospectively test biomarker- or imaging-guided enrichment strategies using hs-CRP, lipoprotein(a), oxidized phospholipids, clonal hematopoiesis, and specific plaque phenotypes [27,28,30,32,35,43,50,54,55,56,57]. Safety monitoring also requires more rigorous prospective characterization, particularly focusing on gastrointestinal intolerance, discontinuation risk, drug–drug interactions, pharmacogenomic susceptibility, and definitively resolving the non-cardiovascular mortality signal [29,31,52,74,129].

## 5. Interpretation and Evidence Value: Synthesizing the Clinical Hierarchy

**Figure 4:**
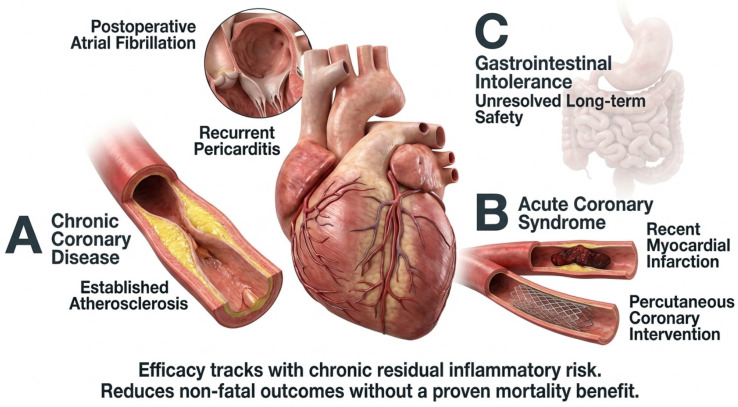
**Visual summary of the Interpretation and Evidence Value of colchicine across cardiovascular settings.** The figure highlights the central interpretive pattern of the evidence: the strongest and most consistent clinical value is seen in chronic CAD and established atherosclerosis, with additional favorable signals in recurrent pericarditis and postoperative AF, whereas evidence is weaker or more inconsistent in ACS, recent MI, and PCI-related settings. It also emphasizes the key safety constraint of gastrointestinal intolerance and the unresolved nature of long-term net safety, while underscoring that the overall benefit is driven primarily by reduction in non-fatal outcomes rather than proven mortality improvement.

Across the full body of syntheses, colchicine has meaningful but conditional cardiovascular relevance (Table 4). Based on the highest tiers of evidence—specifically completed randomized controlled trials and large-scale meta-analyses—the most consistent signal supports its use as an adjunctive anti-inflammatory therapy for residual vascular risk in chronic coronary and broader ASCVD. In this domain, the clinical benefit is concentrated in non-fatal ischemic outcomes—including MI, stroke, coronary revascularization, and composite MACEs—rather than in clearly improved overall survival [7,13,16,34,36,53,84,91,92]. This overarching interpretive pattern, summarized visually in Figure 4, is strongly reinforced by foundational mechanistic and in vivo imaging evidence. These lower-tier but biologically critical studies suggest plaque stabilization, attenuation of vascular inflammation, and greater absolute benefit in higher-risk inflammatory phenotypes. Collectively, this strengthens biological plausibility without, by itself, establishing universal clinical utility [22,23,25,27,28,35,50,124]. Consequently, colchicine is best understood as a clinically relevant but selectively useful targeted adjunct rather than a blanket cardiovascular therapy across all indications.

Secondary to chronic CAD, high-quality evidence is also favorable in recurrent pericardial disease and selected perioperative inflammatory settings. Network and pooled data from completed trials support the use of colchicine in recurrent pericarditis and myopericarditis, demonstrating a reduced recurrence-related burden and lower treatment failure rates [12,46]. Similarly, perioperative administration appears highly beneficial for preventing postoperative AF, especially following cardiac surgery and CABG, confirming a reproducible and clinically valuable anti-inflammatory effect in this acute surgical setting [54,86,109,110,121,130]. These domains differ fundamentally from chronic coronary prevention; their clinical value is highly indication-specific and more mechanistically tied to the immediate control of acute inflammatory complications than to long-term atherothrombotic event reduction.

Descending the evidence hierarchy, the clinical outcome data becomes notably less secure in ACS, recent MI, PCI-related interventions, and acute reperfusion settings (Table 4). While some trial syntheses and observational studies suggest a benefit for selected outcomes, such as stroke, urgent revascularization, or left ventricular recovery [32,57,87,125], the broader landscape of completed randomized trials, subsequent re-analyses, and real-world studies yields conflicting results. Many of these robust studies found no clear reduction in infarct size, myocardial injury, major cardiovascular events, or longer-term clinical outcomes when colchicine was initiated acutely following an ACS event [39,44,51,55,56,74,75,79,89,131,138,140]. Taken together, these contrasting findings indicate that the drug is currently more convincingly supported as a longer-term secondary prevention strategy than as a short-course intervention in acute ischemic care [13,55,90,91].

Outside of classic coronary and pericardial prevention, applications in other cardiovascular domains remain largely supported by preliminary, observational, or mechanistic data rather than definitive, completed Phase III trials. Findings in peripheral arterial disease (PAD) are promising but primarily observational and not yet definitive. Concurrently, its application in heart failure and nonischemic cardiomyopathy remains supported predominantly by mechanistic insights, pilot data, or mixed evidence rather than settled, large-scale clinical outcome data [15,47,71,101,116].

The overall interpretive weight of this evidence base is fundamentally shaped by tolerability and safety constraints. The most consistent adverse-effect signal across all levels of evidence is gastrointestinal intolerance, particularly diarrhea, abdominal symptoms, and subsequent treatment discontinuation [38,52,54,84,86,109,110,130,139] (Table 4). In addition, some meta-analyses and trial data raise ongoing concerns regarding an increase in non-cardiovascular mortality; however, this signal remains statistically inconsistent across completed studies and is therefore categorized as unresolved rather than definitive [31,74,106,107,129,134], as the most recent meta-analysis reported neutral effects of colchicine on non-cardiovascular deaths (risk ratio 1.08, 95% CI 0.76–1.54) [85]. Furthermore, known drug–drug interaction concerns, particularly with CYP3A4 and P-glycoprotein inhibitors, complicate its routine real-world use in patients requiring multiple cardiovascular polypharmacy regimens [129].

Recognizing the current limitations of this evidence base is critical for contextualizing its value. The primary limitation is the substantial heterogeneity in clinical indication, treatment timing, comparator structure, and follow-up duration across the literature. There is also a recurrent disconnect between observed biomarker or mechanistic improvements and hard clinical outcomes, especially in PCI, STEMI, and acute coronary settings [39,44,51,55,56,75,131,136,138,140]. Consequently, the literature is uneven in evidentiary maturity: strong pooled data solidifies its role in chronic CAD, whereas much weaker support characterizes PAD, heart failure, nonischemic cardiomyopathy, and several periprocedural applications where observational, mechanistic, imaging, protocol, or pilot studies still predominate [9,11,14,15,17,20,22,23,47,71,101,116]. Furthermore, the safety interpretation remains incomplete; while gastrointestinal intolerance is consistently confirmed, the signal for non-cardiovascular mortality remains unresolved [31,52,74,84,107,129].

To resolve these gaps, future and ongoing research must prioritize large, indication-specific randomized trials designed to explicitly distinguish chronic secondary prevention from acute coronary, periprocedural, heart failure, PAD, and nonischemic inflammatory uses. These prospective studies must also clarify optimal treatment timing, cumulative exposure limits, and biomarker-guided patient selection, particularly focusing on phenotypes with higher residual inflammatory risk [9,10,13,14,17,32,35,47,71,98,112,113]. Ultimately, until these ongoing questions are resolved through stronger randomized confirmation, colchicine’s net benefit remains inherently indication-dependent, requiring careful patient selection and diligent longer-term safety assessment.

## 6. Evidence-to-Practice Roadmap for Colchicine in Cardiovascular Disease

Table 5 provides an evidence-to-practice roadmap for the use of colchicine in the management of cardiovascular diseases. The field has fundamentally moved beyond asking whether colchicine works in cardiovascular disease in general; the real research task is to define where it works, in whom, when to start, how long to continue, and which biological signals justify its use. New applications remain limited by heterogeneity, incomplete clinical confirmation, or unresolved safety and implementation questions. This summary matrix synthesizes the field from established biological and clinical evidence down to unresolved translational and practice questions. Future progress depends on indication-specific, adequately powered randomized trials, standardized intervention frameworks, biomarker- and imaging-guided enrichment, and more rigorous long-term safety characterization.

## 7. Conclusions: Defining the Therapeutic Scope

The current evidence base supports colchicine not as a universal cardiovascular therapy, but as an indication-dependent anti-inflammatory adjunct with uneven maturity across clinical domains. Based on the highest hierarchy of clinical data, its most defensible role lies in chronic coronary and atherosclerotic disease, where completed randomized evidence is strongest and reductions in recurrent non-fatal ischemic events are most consistent. Additional value is robustly demonstrated in recurrent pericarditis and in preventing postoperative atrial fibrillation. In contrast, applications in acute myocardial infarction, STEMI, PCI-related intervention, heart failure, and several emerging inflammatory applications rely on lower-tier evidence characterized by heterogeneity, smaller observational datasets, or incomplete clinical confirmation. Future progress depends on ongoing trials utilizing more standardized intervention frameworks, clearer endpoint hierarchies, prospective biomarker- and imaging-guided enrichment, and stronger long-term safety assessment. Ultimately, colchicine’s place in cardiovascular care must be defined through precise, syndrome-specific implementation grounded in rigorous trial data, rather than broad therapeutic generalization.

## Figures and Tables

**Table 1:** Cross-study patterns in Population and Study Setup: A Hierarchy of Evidence.

Population/Study-Setup Pattern	Recurring Population Definition	Recurrent Structural Features	Quantitative Setup Markers	Reference(s)
High-Tier Evidence (Completed Parent Trials): Large chronic/post-MI coronary parent trials repeatedly served as source populations for secondary analyses.	Completed chronic coronary disease or recent MI populations were repeatedly reused for subgroup, biomarker, mortality, and treatment–timing analyses.	Randomized parent trials with subsequent secondary, post hoc, or sub-study analyses.	LoDoCo2 enrolled 5522 patients and generated sub-analyses in 1007 patients with T2DM, 1777 biomarker-profiled participants, 854 participants with 2047 observations, 278 EV-profiled patients, and 151 CT angiography participants. COLCOT contributed 4661 post-MI patients, including TTI strata of 1193, 720, and 2748, and a pooled post-MI analysis of 429 patients from COLCOT/LoDoCo-MI.	[25,26,27,28,29,30,31,32,33,34,35,36]
High-Tier Evidence (Major RCTs): Multicenter blinded randomized cardiovascular trials commonly enrolled mid-sized to very large interventional cohorts.	Populations included chronic inflammatory cardiomyopathy, STEMI, post-MI, PCI, acute heart failure, and thoracic surgery patients.	Multicenter, randomized, blinded designs were repeatedly used; several were placebo-controlled, and some were event-driven or factorial.	Planned/enrolled sample sizes were 80 in the planned CMP-MYTHiC, 194 in the completed COVERT-MI, 278 in the ongoing COLICA across 12 sites, 2770 in the ongoing COL BE PCI, 3200 in the planned COP-AF across 40 sites in 11 countries, and 7062 in the completed CLEAR trial across 104 centers in 14 countries.	[6,8,9,10,11,24]
Procedural and Pilot Studies: PCI- and ablation-based studies repeatedly used early follow-up windows and compact procedural cohorts.	Recurrent populations were patients undergoing PCI/PPCI or catheter ablation for AF.	Randomized pilot trials, retrospective comparative cohorts, and dedicated procedural sub-studies were common.	Sample sizes included 75 PCI patients with related 50-patient and 75-patient sub-studies; 321 STEMI patients undergoing PPCI; 714 patients referred for PCI, with 400 treated and a 280-patient biomarker sub-study; 139 radiofrequency ablation patients; 199 AF-ablation patients; 205 HPSD AF-ablation patients; and 1568 ablations in 1412 patients with a 275-per-group matched cohort. Follow-up was commonly 14 days, 30 days, 2 weeks/3 months, or 1 month.	[37,38,39,40,41,42,43,44]
Observational Evidence: Propensity-matched or matched retrospective cohorts were recurrent in nonrandomized cardiovascular settings.	TAVI, post-ablation arrhythmia, myopericarditis, and lower-limb PAD were repeatedly studied using matched comparisons.	Matching rather than randomization defined the comparison structure.	Matched sets included 705 colchicine-exposed versus 702 controls after TAVI, 90 versus 90 catheter-ablation patients, 73 matched myopericarditis pairs derived from 175 patients, and 52,350 PAD user/non-user pairs with 10-year follow-up.	[45,46,47,48,49]
Mechanistic/Imaging Trials: Coronary and structural heart imaging studies repeatedly selected smaller mechanistic cohorts with serial imaging follow-up.	Stable CAD, ACS/NSTEMI, moderate aortic stenosis, inflammatory cardiomyopathy, and STEMI populations were selected for assessment of anatomical or tissue characterization.	Serial CCTA, OCT, CT angiography, PET-CT, CMR, FDG-PET, and echocardiography were embedded in the trial design.	Imaging-focused cohorts included 84 EKSTROM participants with baseline/12-month CCTA, 64 COCOMO-ACS participants randomized 1:1 with serial OCT over 18 months, 128 ACS patients in COLOCT with 12-month OCT follow-up, 150 CHIANTI participants with baseline imaging and 24-month follow-up after a 2-week run-in, 80 planned CMP-MYTHiC patients diagnosed by CMRI or FDG-PET, 151 LoDoCo2 CT-sub-study participants, and 192 first-STEMI patients assessed by CMR at 5 days and 3 months.	[6,8,14,17,18,25,50,51]
Evidence Syntheses: Evidence synthesis frequently aggregated very large, randomized datasets rather than isolated single trials.	Most pooled populations were CAD, ACS/post-MI, PCI, secondary vascular prevention, postoperative AF, or recurrent pericarditis cohorts.	Systematic reviews, conventional meta-analyses, network meta-analyses, cumulative-dose meta-analyses, and pooled IPD analyses recurred.	Pooled sample sizes were 20,601 across 16 RCTs, 14,188 across 21 RCTs, 22,532 across 10 randomized trials, 30,659 across 9 randomized trials, 31,397 across 14 RCTs, and 30,808 across 11 RCTs; more focused syntheses included 12,602 across 3 ACS trials, 13,245 across 5 recent-MI trials, 5540 across 2 post-ACS trials, and 2274 across 12 postoperative AF trials.	[5,7,13,16,52,53,54,55,56,57]
Preclinical/Translational Models: Preclinical cardiovascular evidence repeatedly used small, controlled animal models and short, predefined observation periods.	Experimental populations modeled MI, coronary microembolization, aneurysm biology, myocarditis, atherosclerosis, perioperative myocardial injury, and dilated cardiomyopathy.	Controlled animal experiments, often with parallel treated/control groups, were a recurring architecture.	Sample sizes included 27 pigs (14 vs. 13), 20 rabbits randomized to colchicine/placebo, 40 rats in four groups, 32 mice per group in post-MI work, 28 versus 29 mice in AAA progression, and 46 versus 42 mice after LAD ligation; explicit follow-up windows included 30 days, 80 days, 7 days, 28 days, and 3 days after surgery in the sterile pericarditis model.	[58,59,60,61,62,63,64]
Lower-Tier/Emerging Clinical Evidence: Non-coronary and targeted inflammatory populations were usually studied in smaller, more selective cohorts than coronary secondary-prevention trials.	These populations included HFpEF, recurrent pericarditis, post-Fontan children, type 2 diabetes with coronary calcification, and acute heart failure.	Cross-sectional, pilot, open-label, crossover, observational, and factorial designs were more common than very large, blinded efficacy trials.	Cohort sizes were 154 in the ViKCoVaC factorial trial, 40 HFpEF patients in a crossover trial with two 12-week phases and a 2-week washout, 256 recurrent-pericarditis patients in a retrospective multicenter cohort, 15 treated plus 15 matched controls in refractory pericarditis, and pediatric Fontan populations of 9 (ITT) and 5 (per protocol) versus 25 controls.	[24,65,66,67,68,69,70,71,72]

**Table 2:** Cross-reference patterns in the Intervention and Outcome Framework: Structural Hierarchy.

Intervention/Outcome Pattern	Recurring Intervention Architecture	Core Comparator Structure	Recurrent Endpoint Framework and Technical Specifications	Reference(s)
High-Tier Clinical Efficacy: Long-term low-dose secondary prevention in coronary disease.	Completed large-scale trials utilizing 0.5 mg/day colchicine, typically added to standard therapy; follow-up reported at 12, 18, and 24 months, or event-driven long-term follow-up.	Placebo or standard care.	Composite cardiovascular endpoints centered on MACE/MACCE or time-to-first event, typically comprising CV death, MI, stroke, and coronary/ischemia-driven revascularization.	[7,10,11,34,36,91,92]
In Vivo Mechanistic/Imaging: Serial imaging trials using fixed low-dose colchicine.	Completed imaging sub-studies and trials utilizing 0.5 mg/day colchicine, with repeated imaging over 12 months, 18 months, or 24 months.	Placebo (or placebo/control within a randomized design).	Imaging-defined progression/stabilization endpoints: low-attenuation plaque volume, minimal fibrous cap thickness, total atheroma volume, remodeling index, aortic valve calcium score, aortic valve 18F-NaF uptake, and peak aortic jet velocity.	[14,17,18,50]
Acute/Procedural Settings (Mixed Evidence): Loading-dose/short-course peri-PCI or reperfusion regimens.	Completed and ongoing trials testing preprocedural or reperfusion-linked dosing with explicit loading strategies: 2 mg bolus then 0.5 mg twice daily for 5 days; 1 mg then 0.5 mg 6–24 h pre-PCI; 1.8 mg single preprocedural dose; 1 mg before PPCI then 0.5 mg daily until discharge.	Placebo or no colchicine/standard care.	Acute injury and procedural endpoints: infarct size by CMR at 5 days and/or 3 months, microvascular obstruction, PCI-related myocardial injury, hs-troponin-I change at 24 h, no-reflow, coronary flow reserve, and resistive reserve ratio.	[8,39,40,41,44,51]
Acute/Procedural Settings (Postoperative/Ablation): Short periprocedural prophylaxis around ablation or surgery.	Time-limited regimens clustered around procedures: 0.6 mg twice daily for 7 days after ablation; 0.6 mg twice daily for 10 days after thoracic surgery; 0.6 mg twice daily for 14 days after AF ablation; 500 μg 4 h before CABG then twice daily for 10 days; or colchicine from 7 days before to 1 month after ablation.	Placebo, standard care, no colchicine, or alternative timing strategy.	Procedure-related inflammatory and rhythm outcomes: clinical pericarditis within 14 days, acute pericarditis, perioperative AF/flutter, postoperative AF, AF recurrence, pericardial effusion, pericarditis pain/chest pain, ECG changes, and constrictive physiology at 1 and 4 weeks; one AF study defined recurrence as AF detection >30 s after a 3-month blanking period.	[9,37,38,43,77,108,109,110,111]
Clinical Biomarker Frameworks: Biomarker-centered anti-inflammatory monitoring.	Fixed daily regimens (0.5 mg/day, 0.6 mg/day, 0.5 mg twice daily) or PCI-linked dosing with serial laboratory sampling.	Placebo, standard care, or usual care.	Recurrent biomarker outcomes included hs-CRP, IL-6, IL-1β, IL-10, IFN-γ, TNF-α, WBC/neutrophils, NT-proBNP, sST2, and microRNA profiles; operational thresholds included hs-CRP ≥ 2 mg/L for high inflammation and hs-CRP ≤ 1.0 mg/L as a target post-treatment level.	[24,28,30,42,71,112,113,114,115]
Observational/Epidemiological Evidence: PAD/limb-event prevention frameworks extending beyond coronary composites.	Observational cohort exposure evaluated as user vs. non-user, or long-term vs. short-term use, spanning follow-ups from 2 years to 10 years.	NSAIDs, non-use, or matched control cohorts.	Expanded event structure: major adverse limb events (MALEs), major adverse cardiovascular and limb events, major amputations/any lower-limb amputation, revascularization for limb ischemia, plus MACEs and death.	[47,78,116]
Preclinical/Translational Frameworks: Concentration-based mechanistic systems in ex vivo/in vitro studies.	Explicit nonclinical exposure ranges: 10 μM, 25 nmol/L, 2 ng/mL, 10 ng/mL, or 0.05–5 mg/mL/g cholesterol; exposure windows included 90 min and 24 h.	Vehicle or untreated control.	Mechanistic readouts (rather than clinical composites): maximal platelet aggregation, GPVI signaling, NET release/stimulated NET formation, tubulin organization, tissue factor expression and FXa generation, force–length work loops, end-diastolic stiffness, collagen degradation, macrophage proliferation, myofibroblast activation, and cholesterol-crystal volume/morphology.	[19,20,21,22,23,103,104]
Emerging Clinical Indications: Heart failure/cardiomyopathy studies using divergent endpoints despite recurrent colchicine use.	Completed pilot and ongoing trials using regimens ranging from 0.5–1.0 mg/day to 0.5 mg twice daily, with a 2-mg loading dose followed by 0.5 mg twice daily for 8 weeks.	Placebo, usual care, standard care, vehicle, or saline.	Heterogeneous endpoint architecture: 6-month composite of clinical events + PVC burden increase ≥50% + LVEF reduction >10%; time-averaged NT-proBNP change to week 8; sST2 and hsCRP at 12 weeks; HDRS-21 over 12 weeks; and experimental measures of cardiac function, fibrosis, cytokines, neutrophil recruitment, and NLRP3-related signaling.	[6,24,71,101,117]

**Table 3:** Cross-study evidence patterns for Findings: A Hierarchy of Outcomes.

Specific Findings Pattern	Cross-Study Quantitative Signal	Key Contrast/Boundary of the Pattern	Reference(s)
High-Tier Clinical Efficacy: Chronic/stable coronary and atherosclerotic disease: recurrent reduction in non-fatal ischemic events	Completed pooled analyses showed lower MACEs: RR 0.67 (95% CI 0.56–0.80), RR 0.73 (95% CI 0.57–0.95), RR 0.83 (95% CI 0.73–0.95), RR 0.65 (95% CI 0.52–0.82), RR 0.64 (95% CI 0.51–0.80). MI rate was reduced in several analyses: RR 0.74 (95% CI 0.59–0.93); RR 0.76; RR 0.83 (95% CI 0.72–0.96); RR 0.78; RR 0.73 (95% CI 0.55–0.98). Revascularization was also reduced: RR 0.72 (95% CI 0.53–0.99), RR 0.61, RR 0.79 (95% CI 0.65–0.94).	Benefit is concentrated on non-fatal ischemic endpoints; effect sizes vary across meta-analyses.	[5,7,16,84,91,92]
High-Tier Efficacy Boundaries: Mortality: generally neutral across pooled coronary disease evidence.	No significant reduction in all-cause mortality or cardiovascular mortality in multiple completed pooled analyses: all-cause death RR 0.97 (95% CI 0.78–1.22), OR 0.93 (95% CI 0.63–1.36), RR 1.01, RR 0.96; cardiovascular death RR 0.98 (95% CI 0.79–1.21), RR 0.73, RR 0.94, RR 0.82.	Neutral mortality findings coexist with non-fatal ischemic benefit.	[5,7,16,84,90,92]
Timing/Sub-study Analyses: Early exposure/intensity may modify the benefit after acute atherothrombotic events.	Initiation within 3 days after MI reduced the primary endpoint (HR 0.52, 95% CI 0.32–0.84), urgent angina hospitalization (HR 0.35), and all coronary revascularization (HR 0.63). Separately, cumulative exposure ≥ 90 mg-days was associated with lower MACEs (OR 0.66, 95% CI 0.52–0.84).	Later initiation after MI was not significant: HR 0.36 (95% CI 0.53–1.75) at 4–7 days and HR 0.82 (95% CI 0.61–1.11) after 8 days.	[13,32]
Mixed Clinical Evidence: ACS/recent MI: overall effect remains inconsistent across randomized evidence	Some favorable signals were observed, including fewer MACEs in one completed ACS trial (8/120 vs. 28/129, *p* = 0.001) and a pooled ACS composite reduction of 5.5% vs. 7.6% (OR 0.67, 95% CI 0.46–0.98), with lower cerebrovascular events (OR 0.31, 95% CI 0.14–0.69).	Several other completed datasets were neutral: recent-MI meta-analysis RR 0.83 (95% CI 0.66–1.04); COPS primary composite 24 vs. 38 (*p* = 0.09); another STEMI trial showed no significant short-term clinical benefit.	[51,55,57,74,76,131,132]
Surgical Prophylaxis (Strong Evidence): Postoperative atrial fibrillation (POAF) prevention: consistent benefit in surgical settings	POAF was reduced across completed pooled and trial-level evidence: RR 0.65 (95% CI 0.56–0.75), RR 0.54 (95% CI 0.40–0.73), OR 0.515 (95% CI 0.281–0.943).	The benefit is surgical/procedural; it does not establish a uniform benefit for all AF-ablation settings.	[54,109,121]
Procedural/Ablation Outcomes (Mixed Evidence): AF ablation/peri ablation outcomes: anti-inflammatory benefit is more reproducible than rhythm-control benefit	Favorable findings included lower long-term AF recurrence in one matched study (HR 0.78, 95% CI 0.63–0.96; propensity-matched HR 0.71, 95% CI 0.53–0.96) and lower acute pericarditis with pre-/post-ablation colchicine (1.9% vs. 17.5%; post-only 7.5%; *p* < 0.001).	Several completed studies found no reduction in short-term arrhythmia recurrence, post-ablation pericarditis, chest pain, effusion, emergency visits, or hospitalization.	[37,38,43,48,108]
Pericardial Disease (Strong RCT/Cohort Evidence): Recurrent pericardial disease: convergent reduction in recurrence and related events	Recurrence was lower in completed pooled and cohort data: RR 0.46 (95% CI 0.37–0.58); treatment failure RR 0.42 (95% CI 0.31–0.57); rehospitalization RR 0.26 (95% CI 0.10–0.70). Combination therapy data showed recurrence rates of 18.8% vs. 31.3% and an HR of 0.52 (95% CI 0.29–0.91). Myopericarditis recurrence was 19.2% vs. 43.8%.	This is one of the most internally consistent clusters of clinical benefits in the evidence base.	[12,46,66]
Observational/Epidemiological Evidence: Peripheral arterial disease/limb-event prevention: favorable pooled and observational signals, but not uniform across designs	Major adverse limb events HR 0.84 (95% CI 0.75–0.94), MACE HR 0.90 (95% CI 0.82–0.98), major amputation HR 0.81, limb revascularization HR 0.81. A large, matched cohort found composite cardiovascular/limb events HR 0.90 (95% CI 0.88–0.92), amputation HR 0.84, ischemic revascularization HR 0.85, and all-cause mortality HR 0.90.	A target-trial emulation in older PAD patients found no conclusive benefit: risk ratio 0.95 (95% CI 0.83–1.07) versus NSAIDs and 0.92 (95% CI 0.70–1.16) for long- vs. short-term colchicine.	[47,78,116]
Clinical Biomarker Frameworks: Inflammatory biomarker attenuation: highly recurrent across clinical and mechanistic studies	hs-CRP reduction pooled as MD −1.59 (95% CI −2.40 to −0.79); odds of hs-CRP ≤ 1.0 mg/L after MI increased (OR 1.64, 95% CI 1.07–2.51). After PCI, IL-6 rose less with colchicine (76% vs. 338%, *p* = 0.02) and hs-CRP rose less (11% vs. 66%, *p* = 0.001). In MACT, hs-CRP declined from 6.1 mg/L to 0.6 mg/L, and hs-CRP ≥ 2 mg/L fell from 81.8% to 11.8% (both *p* < 0.001).	Biomarker improvement did not always translate into lower hard clinical event rates in PCI or reperfusion trials.	[28,30,44,114,133]
In Vivo Imaging/Mechanistic Evidence: Plaque stabilization/remodeling: favorable structural signals, but not all imaging endpoints improve	CT showed a thicker fibrous cap with colchicine (87.2 μm vs. 51.9 μm; difference 34.2 μm, *p* = 0.006), lower lipid arc (−35.7° vs. −25.2°, *p* = 0.004), and less macrophage extension (difference −6.0°, *p* = 0.044). CT-based studies showed lower total atheroma volume (0.3 vs. 1.4, *p* = 0.008) and lower follow-up values (0.3 vs. 1.4, *p* = 0.008); calcified plaque volume was higher in another study (169.6 mm^3^ vs. 113.1 mm^3^, *p* = 0.041).	Coronary calcification activity/Trax and some low-attenuation plaque measures were unchanged in other completed imaging studies.	[25,50,65,124]
Established Safety Profile: Gastrointestinal toxicity: the most consistent adverse-effect pattern	Across 21 completed RCTs, GI events occurred in 16.1% vs. 12.2% (RR 2.16, 95% CI 1.50–3.12), diarrhea in 12.5% vs. 8.1% (RR 2.77, 95% CI 1.55–4.94), and discontinuation in 4.8% vs. 3.4% (RR 1.54). Individual trials reported GI discomfort: 47% vs. 15%; diarrhea: 26% vs. 7%; and abdominal pain: 7.0% vs. 1.6%.	GI harm is consistent across settings and is the most reproducible countervailing finding.	[38,43,52,77,109]
Unresolved Safety Signals: Non-cardiovascular mortality signal: inconsistent and unresolved	Some analyses reported higher non-cardiovascular death: OR 1.54 (95% CI 1.10–2.15), RR 1.53 (95% CI 1.10–2.14), COPS non-cardiovascular death 5 vs. 0 (*p* = 0.024). LoDoCo2 cause-specific analysis showed non-cardiovascular death 1.9% vs. 1.3% (HR 1.51, 95% CI 0.99–2.31).	Other completed pooled analyses showed no significant increase in non-cardiovascular death; so, this pattern remains inconsistent rather than definitive.	[7,31,53,74,85,129,134]

**Table 4:** Cross-reference evidence patterns for Interpretation and Evidence Value: A Hierarchy of Interpretation.

Interpretation/Evidence Pattern	Evidence Cluster	Key Technical/Quantitative Signal	Meaningful Limitation/Contrast	Reference(s)
High-Tier Clinical Efficacy: Secondary prevention benefit is concentrated in non-fatal ischemic events in chronic coronary/atherosclerotic disease	Multiple completed meta-analyses and pooled syntheses consistently report lower MACEs with parallel reductions in MI, stroke, and/or coronary revascularization	MACEs: OR/RR 0.64–0.83; MI: OR/RR 0.73–0.84; stroke: OR/RR 0.47–0.55; revascularization: OR/RR 0.61–0.73	Effect is recurrently driven by non-fatal events rather than mortality	[7,13,16,53,84,91]
High-Tier Efficacy Boundaries: Mortality benefit is not established despite recurrent event reduction	Across completed reviews/meta-analyses, CV death and all-cause death usually remain non-significant even when MACEs fall	All-cause death: RR/OR 0.93–1.01 in pooled analyses; CV death commonly non-significant, including RR 0.94, RR 0.96, and OR 0.82	Supports adjunctive vascular-risk reduction, not proven survival gain	[5,7,16,53,85,90,91,92,136]
Mixed Clinical Evidence: Evidence in ACS/recent MI is directionally favorable in some datasets but overall inconsistent	Some completed ACS/post-MI syntheses report lower composite events or stroke; others report neutral primary outcomes or non-robust MACE effects	Neutral/uncertain results include RR 0.83 (95% CI 0.66–1.04) after recent MI and OR 0.82 (95% CI 0.63–1.07) in acute/recent coronary syndromes; modest ACS MACE reduction RR 0.73 (95% CI 0.54–0.99) lost robustness on sensitivity analysis; positive ACS composite OR 0.67 (95% CI 0.46–0.98) in a 2-trial meta-analysis.	Pattern supports heterogeneity by timing, population, or trial mix rather than stable post-ACS efficacy	[55,56,57,74,87,138,139]
Mechanistic vs. Clinical Translation: PCI/STEMI periprocedural use often changes inflammatory or physiologic markers more than hard outcomes	Completed trials and sub-studies repeatedly show reduced inflammatory biomarkers, neutrophil counts, troponin changes, or improved microvascular physiology, but neutral clinical endpoints in larger PCI/STEMI settings.	Less IL-6/hs-CRP rise after PCI [44]; lower post-PCI hs-troponin-I change and fewer periprocedural injuries [40]; higher post-PCI CFR and resistive reserve ratio [41]; yet no reduction in PCI-related myocardial injury or 30-day events [44], no long-term MACE benefit after single-dose pre-PCI colchicine [140], and no STEMI PPCI benefit for no-reflow, EF, or MACEs [39]	Mechanistic signal is reproducible, but clinical translation is inconsistent.	[22,39,40,41,42,44,131,140]
Strong RCT/Cohort Evidence: Recurrent pericarditis/myopericarditis is one of the strongest positive domains	Completed network/meta-analytic and observational evidence converges on fewer recurrences and lower treatment burden	Recurrence RR 0.46 (95% CI 0.37–0.58); treatment failure RR 0.42; rehospitalization RR 0.26; observational studies also report lower recurrence and longer event-free survival	More favorable and consistent than most non-coronary cardiovascular indications	[12,46,66,122]
Surgical Prophylaxis (Strong Evidence): Postoperative atrial fibrillation prevention is a repeatable positive signal, especially after cardiac surgery/CABG	Completed randomized trials and pooled analyses show lower POAF/AF incidence after surgery	RR 0.54 after CABG [121]; RR 0.65 across postoperative settings [54]; RR 0.75 in updated AF prevention meta-analysis [130]; OR 0.515 in the COCS trial [109]	Benefit is offset by more GI toxicity and is less clearly established outside cardiac surgical settings	[54,86,109,121,130]
Established Safety Profile: Gastrointestinal toxicity is the dominant recurring adverse-effect pattern	Safety meta-analyses and individual completed trials consistently show more diarrhea, GI discomfort, abdominal pain, and discontinuation	GI events RR 2.16; diarrhea RR 2.77; discontinuation RR 1.54 [52]; GI adverse events RR 1.67 with discontinuation RR 1.54 [84]; excess diarrhea/abdominal pain also reported in RCTs and periprocedural studies [38,43,86,109]	This is the most consistent practical constraint on net benefit	[38,43,52,84,86,109,110,130]
Unresolved Safety Signals: Non-cardiovascular mortality remains an unresolved safety signal rather than a settled harm.	Some completed pooled analyses and trials report excess non-CV death, whereas others do not confirm a significant increase.	on-CV death OR 1.54 in one meta-analysis [134]; higher non-CV death in COPS [74]; RR 1.53 in safety meta-analysis [129]; comparator network meta-analysis reported RR 1.48 vs. NPC1L1 inhibitors and RR 1.57 vs. PCSK9 inhibitors [107]; by contrast, LoDoCo2 cause-of-death analysis found no treatment association with specific causes of death [31], and pooled analyses reported no significant non-CV mortality increase [16,91]	Safety interpretation remains uncertain, limiting unequivocal routine endorsement.	[16,31,74,91,107,129,134]
Targeted Clinical Positioning: Evidence supports risk-targeted and exposure-dependent use, not indiscriminate use.	Completed sub-analyses suggest consistent relative benefit across risk strata, with greater absolute benefit in higher-risk/inflammation-enriched groups and after sustained exposure.	Absolute 10-year risk reduction 4.6% and 2.0 MACE-free life-years gain in individual-risk modeling [98]; cumulative exposure threshold ≥90 mg-days associated with benefit [13]; higher absolute benefit with elevated Lp(a) (4.4% vs. 2.4%) and significant interaction in highest OxPL-apoB tertile [35]; benefit consistent across baseline risk groups and prior ACS timing [34,36]	Supports selective deployment in chronic residual inflammatory risk rather than universal use in all patients	[13,34,35,36,98]
Health Economics & Modeling: Economic value is favorable where clinical efficacy is already supported	Cost-effectiveness models derived from completed post-MI, chronic coronary disease, and post-CABG trial populations consistently favor colchicine	Dominant vs. standard care after MI in Canadian models [93,96]; €12,176/QALY societal and €19,499/QALY healthcare-perspective in chronic coronary disease [94]; post-CABG ICER $26,684/QALY with median Monte Carlo ICER $19,598 [95]	These are model-based results and depend on the validity of underlying efficacy assumptions	[93,94,95,96]

**Table 5:** Evidence-to-Practice Roadmap.

What We Know (Current Evidence Base)	What We Do Not Yet Know (Knowledge Gaps)	What Researchers Should Do Next (Future Directions)
Mechanistic Rationale: Persistent low-grade inflammation is a central component of cardiovascular disease biology, including plaque evolution, destabilization, thrombogenicity, recurrent ischemic risk, and procedure-related injury. Colchicine, therefore, has a strong translational rationale in cardiovascular medicine.	The field still lacks clarity on which modifiable inflammatory axis truly drives specific cardiovascular syndromes and, therefore, is most suitable for targeted colchicine-based intervention.	Move from a broad inflammation hypothesis to syndrome-specific inflammatory risk models that define exactly where colchicine is biologically justified.
Breadth of Scope: Colchicine has been investigated across a wide cardiovascular spectrum, including chronic and ACS, post-MI care, revascularization, atrial fibrillation, pericardial disease, heart failure, PAD, and mechanistic translational settings.	Breadth of investigation does not equal uniform evidentiary maturity; many indications remain much less developed than coronary secondary prevention.	Avoid extrapolation across syndromes and prioritize rigorous, independent evidence development for each specific indication.
Hierarchy of Evidence: The most mature trial architecture is concentrated in chronic CAD, ACS, and post-MI settings, especially through large, completed randomized, placebo-controlled, multicenter trials.	Many non-coronary areas still depend on lower-tier evidence, such as smaller observational, pilot, crossover, mechanistic, retrospective, or nested analyses. Some literature is deepened by repeated analyses of the same parent trials rather than by analyses of new populations.	Build new independent multicenter cohorts and prospective randomized trials in underrepresented phenotypes.
Established Efficacy: The clearest benefits are the reduction in recurrent non-fatal ischemic events in chronic coronary and atherosclerotic disease, prevention of recurrent pericarditis, prevention of postoperative atrial fibrillation, and attenuation of inflammatory or plaque-related markers.	Benefit is less consistent in acute coronary syndromes, STEMI, PCI-related hard outcomes, heart failure, and several emerging indications; a definitive mortality benefit is generally not demonstrated.	Focus future trials on indications with unresolved efficacy, especially acute and periprocedural settings, rather than revisiting already established patterns without a new question.
Study Design: Colchicine has been operationalized through recurring design motifs: chronic low-dose prevention, short periprocedural or post-event use, and mechanistic or biomarker-enriched studies.	Although there is no single validated regimen across syndromes; timing, dose, loading strategy, duration, and discontinuation rules remain highly variable, the standard dose for secondary prevention of ASCVD is 0.5 mg once daily.	Standardize regimen architecture and reporting, especially for timing-sensitive settings such as MI, PCI, and ablation.
Outcome Frameworks: Outcome assessments have included clinical events, arrhythmic and procedural complications, biomarkers, imaging surrogates, mechanistic readouts, safety endpoints, and economic analyses.	Cross-study comparability is limited because outcomes are highly heterogeneous, and improvements in biomarkers or mechanisms do not consistently translate into hard clinical benefit.	Develop core outcome sets that seamlessly align surrogate and mechanistic measures with adjudicated clinical endpoints.
Biological Pathways: Mechanistic plausibility includes inflammasome-linked pathways, neutrophil activation, platelet-thromboinflammatory signaling, cytoskeletal regulation, and plaque-level biology.	It remains unresolved which of these mechanisms is dominant in specific clinical syndromes and whether mechanistic changes reliably identify treatment responders.	Prospectively integrate biomarker and imaging enrichment into adequately powered ongoing trials rather than relying mainly on isolated nested analyses.
Safety Profile: Gastrointestinal intolerance is the most consistent and reproducible adverse effect across completed studies.	Long-term safety remains incompletely defined, including discontinuation burden, drug–drug interactions, pharmacogenomic susceptibility, and the unresolved signal for non-cardiovascular mortality.	Strengthen prospective long-term safety evaluation, including treatment persistence, interaction profiles, and tracking of non-cardiovascular outcomes.
Clinical Positioning: The overall evidence supports colchicine as a targeted adjunct, not a universal cardiovascular therapy. Its most defensible role is in chronic coronary and established atherosclerotic disease, with additional value in recurrent pericarditis and selected postoperative inflammatory settings.	The threshold for routine use remains uncertain in acute coronary, periprocedural, heart-failure, PAD, and nonischemic inflammatory contexts.	Translate the field toward precision clinical positioning, defining where colchicine should be adopted, where it should remain investigational, and what level of evidence is required for each indication.

## Data Availability

No new data was created or analyzed in this study. Data sharing is not applicable.
